# Associations of Early Childhood Manganese and Lead Coexposure with Neurodevelopment

**DOI:** 10.1289/ehp.1003300

**Published:** 2011-09-01

**Authors:** Birgit Claus Henn, Lourdes Schnaas, Adrienne S. Ettinger, Joel Schwartz, Héctor Lamadrid-Figueroa, Mauricio Hernández-Avila, Chitra Amarasiriwardena, Howard Hu, David C. Bellinger, Robert O. Wright, Martha María Téllez-Rojo

**Affiliations:** 1 Department of Environmental Health, Harvard School of Public Health, Boston, Massachusetts, USA; 2 Department of Developmental Neurobiology, Instituto Nacional de Perinatologia, Mexico City, Mexico; 3 Center for Perinatal, Pediatric and Environmental Epidemiology, Department of Epidemiology and Public Health, Yale University School of Medicine, New Haven, Connecticut, USA; 4 Channing Laboratory, Department of Medicine, Brigham and Women’s Hospital, Harvard Medical School, Boston, Massachusetts, USA; 5 Division of Statistics, Center for Evaluation Research and Surveys, National Institute of Public Health, Cuernavaca, Morelos, Mexico; 6 Ministry of Health, Mexico City, Mexico; 7 Department of Environmental Health Sciences, University of Michigan School of Public Health, Ann Arbor, Michigan, USA; 8 Department of Neurology, Harvard Medical School and Children’s Hospital Boston, Boston, Massachusetts, USA; 9 Department of Emergency Medicine, Children’s Hospital Boston, Boston, Massachusetts, USA

**Keywords:** coexposure, early childhood, lead, manganese, metals, neurodevelopment

## Abstract

Background: Most toxicologic studies focus on a single agent, although this does not reflect real-world scenarios in which humans are exposed to multiple chemicals.

Objectives: We prospectively studied manganese–lead interactions in early childhood to examine whether manganese–lead coexposure is associated with neurodevelopmental deficiencies that are more severe than expected based on effects of exposure to each metal alone.

Methods: Four hundred fifty-five children were enrolled at birth in an longitudinal cohort study in Mexico City, provided blood samples, and were followed until 36 months of age. We measured lead and manganese at 12 and 24 months and assessed neurodevelopment at 6-month intervals from 12 to 36 months of age using Bayley Scales of Infant Development–II.

Results: Mean (± SD) blood concentrations at 12 and 24 months were, respectively, 24.7 ± 5.9 μg/L and 21.5 ± 7.4 μg/L for manganese and 5.1 ± 2.6 μg/dL and 5.0 ± 2.9 μg/dL for lead. Mixed-effects models, including Bayley scores at five time points, showed a significant interaction over time: highest manganese quintile × continuous lead; mental development score, β = –1.27 [95% confidence interval (CI): –2.18, –0.37]; psychomotor development score, β = –0.92 (95% CI: –1.76, –0.09). Slopes for the estimated 12-month lead effect on 18-month mental development and 24- through 36-month psychomotor development scores were steeper for children with high manganese than for children with midrange manganese levels.

Conclusions: We observed evidence of synergism between lead and manganese, whereby lead toxicity was increased among children with high manganese coexposure. Findings highlight the importance of understanding health effects of mixed exposures, particularly during potentially sensitive developmental stages such as early childhood.

Most toxicologic studies focus on a single agent and either do not measure or do not adjust for potential confounding or modifying effects of other chemicals. Although such an approach has identified toxicities associated with various chemicals, it does not reflect real-world scenarios in which humans are exposed to multiple chemicals (Cory-Slechta 2005; Simmons 1995). Human exposure to chemical mixtures is particularly widespread in socioeconomically disadvantaged populations (Naess et al. 2007) and among populations living near hazardous waste sites (Hu et al. 2007). The customary approach of examining chemicals in isolation may constrain our ability to understand neurologic sequelae (Bellinger 2008a; Cory-Slechta et al. 2008). Despite the importance of examining joint exposures to toxicants, few epidemiologic studies have done so. Metals, in particular, are common neurotoxicants occurring within the environments of children. Concomitant exposure to several metals may have more severe (i.e., synergistic) effects on cognition than expected based on effects of exposure to each metal alone (Wright et al. 2006).

In this article, we focus on interactions between lead and manganese because these metals share the central nervous system as their primary toxicity target in children. Adverse effects of lead on neurodevelopment are well described (Bellinger et al. 1991; Canfield et al. 2003; Chen et al. 2007; Tellez-Rojo et al. 2006). Cognitive deficits are major concerns, with declines of about 1.1–3.9 IQ points, for example, estimated for a 10-μg/dL increase in blood lead (Lanphear et al. 2005). Evidence for the neurotoxic effects of environmental manganese on children is mounting and includes associations with cognition, memory, behavior, and motor function (Bouchard et al. 2007, 2011; Claus Henn et al. 2010; Menezes-Filho et al. 2011; Riojas-Rodriguez et al. 2010; Takser et al. 2003; Wasserman et al. 2006; Wright et al. 2006). The mechanisms for lead neurotoxicity include increased spontaneous release of neurotransmitters and disruption of calcium metabolism (Bressler and Goldstein 1991). Biological mechanisms for manganese toxicity are also hypothesized to involve calcium metabolism, oxidative damage to neuronal cells, and impaired dopamine neurotransmission (Aschner and Aschner 1991; Normandin and Hazell 2002; Verity 1999). With the same target organ, potentially similar biological mechanisms, and a realistic potential for joint exposure, manganese and lead are important chemicals to examine for interactive effects.

Our study examined manganese–lead interaction in early childhood, a period of potentially heightened susceptibility to neurotoxicants. Among 1- to 3-year-old children in Mexico City, we prospectively examined whether coexposure to manganese and lead causes neurodevelopmental deficiencies that are more severe than expected based on effects of exposure to each metal alone.

## Materials and Methods

*Study population.* The population of children analyzed in this study has been described previously (Claus Henn et al. 2010). Briefly, we enrolled women just before or during pregnancy (*n* = 462) or at delivery (*n* = 462) from 1997 through 2000 in a larger study examining plasma lead biomarkers during pregnancy and neurodevelopment in Mexico City (Surkan et al. 2008; Tellez-Rojo et al. 2006). For the present study, we included 493 children who provided a blood sample at 12 and/or 24 months of age (12 months, *n* = 301; 24 months, *n* = 482) and underwent neurodevelopmental testing. Children were followed through 36 months of age. The parent study for this project did not include measures of blood manganese, but whole blood was archived during follow-up visits whenever possible. After exclusions [e.g., very low birth weight (< 1,500 g), severely premature birth (< 32 weeks gestation), or missing data on gestational age], 486 children (12 months, *n* = 296; 24 months, *n* = 475) met eligibility criteria.

Eligible mothers were informed about the study, and written consent was obtained before participation. The human subjects committees of the National Institute of Public Health of Mexico, Harvard School of Public Health, and participating hospitals approved all study materials and procedures.

*Measurement of blood manganese and lead.* Venous whole blood samples were collected in trace metal-free tubes from children at 12 and/or 24 months of age and immediately frozen. We prepared and analyzed samples for manganese and lead concentrations at the Trace Metals Laboratory at Harvard School of Public Health in Boston. We measured metals concentrations with a dynamic reaction cell/inductively coupled plasma mass spectrometer (Elan 6100; PerkinElmer, Norwalk, CT, USA), using previously described methods and quality control measures (Zota et al. 2009). Recovery rates for manganese and lead in quality control and spiked samples were 78–113%, and precision (percent relative standard deviation) was < 5%. The average limits of detection were 0.09 and 0.04 μg/dL for manganese and lead, respectively, and all samples were above the limits of detection.

*Measurement of child development and potential confounders.* We assessed child neurodevelopment at 6-month intervals (i.e., 12, 18, 24, 30, 36 months of age) using the Bayley Scales of Infant Development–II, Spanish version (BSID-IIS) (Bayley 1993). Trained study personnel, who were blinded to children’s manganese and lead levels, administered the test using a standardized protocol previously described by our research group (Gomaa et al. 2002). In this study, we considered scores from the Mental Development Index (MDI) and the Psychomotor Development Index (PDI) of the BSID-IIS as the primary outcomes.

We collected information on demographic, socioeconomic, and other factors that could confound the relationship between manganese–lead and neurodevelopment: sex, hemoglobin at 12 and 24 months, ferritin at 12 and 24 months, birth weight, birth length, head circumference at birth, gestational age, maternal age at delivery, maternal and paternal education, maternal marital status at enrollment, duration of breast-feeding in the infant’s first year, and child nutrition at 12 and 24 months collected via questionnaires. Umbilical cord blood lead and maternal blood lead at 1-month postpartum were also considered as confounders, in order to examine effects of metals at 12 and 24 months independently from effects of exposures at birth. We assessed maternal IQ using the Wechsler Adult Intelligence Scale, Spanish version (Wechsler 1968).

*Statistical analyses.* Our previous analysis of the main effect of manganese suggested an inverted U-shaped association between manganese and neurodevelopment at 12 months, whereby lower MDI scores were observed at the lowest and highest quintiles of the manganese distribution compared with midrange levels of manganese (Claus Henn et al. 2010). We therefore examined the manganese–lead interaction at both the lowest and highest manganese quintiles (i.e., highest quintile manganese × lead and lowest quintile manganese × lead, compared with middle manganese quintiles). We modeled lead as a continuous variable, centered at the arithmetic mean value of the distribution. Although manganese and lead data were moderately skewed, the Gauss–Markov model requires that only the model residuals be normally distributed. Because this assumption was satisfied, we did not log transform the exposure variables. Skewness may also arise from the presence of outliers; the influence of identified outliers on effect estimates was examined in sensitivity analyses, as described below. Neurodevelopment scores were approximately normally distributed and were modeled as continuous outcomes.

We fit two (i.e., one for each exposure time point) linear mixed-effects models with repeated measures of Bayley scores to examine the manganese–lead interaction over time. Additionally, we fit separate adjusted regression models for each exposure time point with each Bayley assessment (i.e., 12-month blood metals with 12-, 18-, 24-, 30-, and 36-month Bayley scores; 24-month blood metals with 24-, 30-, and 36-month Bayley scores). We adjusted all models for the same set of covariates described previously (Claus Henn et al. 2010): sex, hemoglobin (grams per deciliter; modeled as continuous variable), gestational age (weeks; continuous variable), maternal IQ (continuous variable), and maternal education (years; continuous variable). We included known predictors of neurodevelopment or strong potential confounders (sex, maternal IQ, and education) *a priori* in multivariable regression models, based on biological plausibility. To the model with *a priori* covariates, we added, one at a time, covariates that were associated in bivariate models (*p* < 0.10) with exposures and outcomes at any time point. Additional covariates were included in the final model if the manganese or lead effect estimates changed > 10%.

Although only ≤ 8% of eligible children (12 months, 21 of 296; 24 months, 38 of 475) were excluded because of missing covariate data, we conducted sensitivity analyses to evaluate the appropriateness of conducting a complete case analysis by comparing crude estimates for manganese–lead interaction among all eligible children (including those missing data) with those for participants with complete data. We also examined the influence of extreme observations, identified with the generalized extreme studentized deviate many-outlier procedure (Rosner 1983), by fitting models with and without outliers.

We conducted statistical analyses using SAS (version 9.2; SAS Institute, Inc., Cary, NC, USA) and R (version 2.7.1; R Foundation for Statistical Computing, www.r-project.org). We considered results statistically significant at *p* < 0.05.

## Results

*Participant characteristics.* From among 486 children who met eligibility criteria (12-month blood metals, *n* = 296; 24-month blood metals, *n* = 475), 455 had complete covariate data (12 months, *n* = 275; 24 months, *n* = 437) and were included in all analyses. More blood samples were collected at 24 months than at 12 months because these were convenience samples that depended on availability of a phlebotomist. Children with blood metals at 12 months were largely a subset of the children with blood metals data at 24 months and appeared similar on sociodemographic characteristics [see Supplemental Material, Table 1 (http://dx.doi.org/10.1289/ehp.1003300)]. In Table 1, we present major characteristics of children included in the analyses. Children included were similar to those who were excluded on most variables. Children who provided a blood sample at a single time point appeared similar on major characteristics to those who provided blood samples at both time points (data not shown).

**Table 1:** Sociodemographic characteristics of study participants and nonparticipants (mean ± SD or %).

Characteristic	Children included in analyses*a*	Nonparticipants*b*
Sex (% male)		50.6		44.2
Birth weight (kg)		3.1 ± 0.4		3.1 ± 0.5
Estimated gestational age (weeks)		38.9 ± 1.3		38.4 ± 2.1
Birth length (cm)		49.8 ± 2.2		49.5 ± 3.1
Head circumference at delivery (cm)		34.1 ± 1.6		34.0 ± 1.6
Blood manganese (μg/L)				
12 months*c*		24.7 ± 5.9		—
24 months*d*		21.5 ± 7.4		—
Blood lead (μg/dL)				
Umbilical cord*e*		4.7 ± 3.1		4.8 ± 2.6
12 months*c*		5.1 ± 2.6		—
24 months*d*		5.0 ± 2.9		—
Hemoglobin (g/dL)				
12 months		11.8 ± 1.3		11.8 ± 1.3
24 months		12.6 ± 1.1		12.1 ± 1.4
Bayley MDI Score (36 months)*f*		93.2 ± 9.1		93.1 ± 8.9
Bayley PDI Score (36 months)*f*		95.8 ± 10.9		95.6 ± 11.5
Maternal marital status (% married)		72.6		75.9
Maternal IQ		88.0 ± 13.0		89.0 ± 11.7
Maternal education (years)		10.7 ± 2.8		11.2 ± 3.6
Maternal age at delivery (years)		25.8 ± 5.3		27.8 ± 5.4
Maternal whole-blood lead (μg/dL)*g*		7.5 ± 4.6		7.3 ± 4.2
**a***n *= 455 except where noted. **b**Nonparticipants (*n* = 165) are children who did not provide a blood sample (*n* = 127) or Bayley score (*n* = 2), had incomplete covariate data (*n* = 33) or inadequate blood data (*n* = 1), or were low gestational age (< 32 weeks, *n* = 2). **c***n* = 275. **d***n* = 437. **e***n* = 290. **f**Only 36-month Bayley scores are provided, for brevity; scores at other time points are similar. **g**Collected at 1-month postpartum.				

*Blood manganese and lead concentrations.* The arithmetic mean (± SD) concentrations at 12 and 24 months were, respectively, for blood manganese, 24.7 ± 5.9 μg/L (range, 15.3–73.9) and 21.5 ± 7.4 μg/L (range, 5.1–93.5), and for blood lead, 5.1 ± 2.6 μg/dL (range, 1.5–21.4) and 5.0 ± 2.9 μg/dL (range, 1.3–37.2). Concentrations were significantly positively correlated at 12 and 24 months for both blood manganese (Spearman correlation = 0.56, *p* < 0.0001) and blood lead (Spearman correlation = 0.61, *p* < 0.0001). At 24 months, manganese and lead concentrations were also positively, but weakly, correlated (Spearman correlation = 0.14, *p* = 0.004). Lead concentrations were higher among boys than among girls at 12 and 24 months, although the difference was significant only at 24 months (mean ± SD: 12 months, boys, 5.3 ± 2.7 μg/dL; girls, 4.9 ± 2.5 μg/dL; 24 months, boys, 5.3 ± 3.3 μg/dL; girls, 4.7 ± 2.4 μg/dL). We observed no significant sex differences in blood manganese levels. There were negligible differences in mean lead concentrations among manganese quintiles (data not shown).

*Associations of coexposure with mental development.* Twelve-month blood metals. Table 2 summarizes results from adjusted mixed-effects models of 12-month blood metals, which included MDI scores at all five time points. We observed a significant manganese–lead interaction over time for children in the highest quintile of 12-month blood manganese [adjusted β = –1.27 (95% confidence interval [CI]: –2.18, –0.37)]. Given these estimates, we expect MDI scores to decline by 0.07 points per 1-μg/dL increase in lead among children with midrange manganese levels, compared with a decline of 2.23 points (i.e., β_manganese quintile 5_ + β_lead_ + β_manganese quintile 5 × lead_ = –0.89 + –0.07 + –1.27) per 1-μg/dL increase in lead among children with high manganese levels. Results from adjusted regression models of 12-month blood metals with MDI scores at five separate time points are presented in Table 3. A significant manganese–lead interaction was observed only at 18 months for children in the highest quintile of 12-month blood manganese [β = –1.74 (95% CI: –3.00, –0.49)]. At 18 months, MDI scores are expected to decline 0.01 points per 1-μg/dL increase in lead among children with midrange manganese levels, compared with 1.80 points (i.e., –0.05 + –0.01 + –1.74) among children with high manganese levels. Although effect estimates at other time points of MDI were not statistically significant, coefficients were all negative and approached significance at 24 months of age (*p* = 0.07). Furthermore, when we included a three-way interaction term (i.e., lead × highest quintile of manganese × time) in the mixed-effects model to examine whether the manganese–lead interaction changed over time, we observed no time trend [β = –0.07 (95% CI: –0.46, 0.32)].

**Table 2:** Effect estimates from mixed models*a* of 12- and 24-month metal concentrations with repeated measures of Bayley*b* scores [-coefficient (95% CI)].

Exposure	Twelve months*c*	Twenty-four months*d*
MDI				
Manganese (quintile 1*e* vs. quintiles 2–4)		–2.08 (–3.98, –0.19)		–0.34 (–2.35, 1.67)
Manganese (quintile 5*f* vs. quintiles 2–4)		–0.89 (–2.79, 1.00)		0.01 (–1.97, 2.00)
Lead (μg/dL)*g*		–0.07 (–0.39, 0.25)		–0.08 (–0.46, 0.30)
Manganese (quintile 1) × lead		–0.31 (–1.25, 0.62)		–0.02 (–0.98, 0.94)
Manganese (quintile 5) × lead		–1.27 (–2.18, –0.37)		–0.09 (–0.63, 0.45)
PDI				
Manganese (quintile 1 vs. quintiles 2–4)		0.47 (–1.28, 2.22)		–0.22 (–2.08, 1.65)
Manganese (quintile 5 vs. quintiles 2–4)		–0.05 (–1.80, 1.70)		0.06 (–1.77, 1.90)
Lead (μg/dL)		–0.27 (–0.56, 0.02)		–0.18 (–0.53, 0.17)
Manganese (quintile 1) × lead		0.10 (–0.76, 0.97)		0.23 (–0.66, 1.13)
Manganese (quintile 5) × lead		–0.92 (–1.76, –0.09)		0.18 (–0.32, 0.68)
**a**Adjusted for sex, gestational age, hemoglobin, maternal IQ, maternal education, and visit. **b**Effect estimates for 12-month metals are from repeated measures models that included Bayley scores at 12, 18, 24, 30, and 36 months. Effect estimates for 24-month metals are from repeated measures models that included Bayley scores at 24, 30, and 36 months. **c***n* = 275. For MDI, number of observations = 1,330; for PDI, number of observations = 1,329. **d***n* = 437. For MDI and PDI, number of observations = 1,240. **e**Range: 12 months, 15.30–20.16 μg/L; 24 months, 5.06–15.57 μg/L. **f**Range: 12 months, 28.14–73.95 μg/L; 24 months, 25.78–93.53 μg/L. **g**Blood lead centered at mean: 12 months, 5.09 μg/dL; 24 months, 4.98 μg/dL.				

**Table 3:** Interaction coefficients from regression models*a* of 12-month metals and five time points of Bayley scores [β-coefficient (95% CI)].

Outcome	*n*	Manganese (quintile 1)*b* × lead*c*^,d^	Manganese (quintile 5)*e* × lead*c*^,d^
MDI						
12 months		275		–0.11 (–1.42, 1.20)		–0.94 (–2.21, 0.33)
18 months		272		–0.87 (–2.16, 0.42)		–1.74 (–3.00, –0.49)
24 months		273		0.63 (–1.18, 2.44)		–1.62 (–3.36, 0.12)
30 months		260		–0.03 (–1.41, 1.35)		–1.08 (–2.42, 0.26)
36 months		250		0.46 (–0.93, 1.84)		–1.10 (–2.44, 0.24)
PDI						
12 months		275		0.69 (–0.50, 1.89)		0.15 (–1.00, 1.31)
18 months		271		–0.69 (–2.08, 0.71)		–1.20 (–2.45, 0.05)
24 months		273		–0.21 (–1.59, 1.18)		–1.60 (–2.93, –0.27)
30 months		260		0.51 (–1.06, 2.07)		–1.69 (–3.21, –0.17)
36 months		250		–1.12 (–2.82, 0.58)		–1.92 (–3.56, –0.28)
**a**Adjusted for sex, gestational age, hemoglobin, maternal IQ, and maternal education. Manganese and lead included as main effects. **b**Range, 15.30–20.16 μg/L. **c**Blood lead variable centered at mean: 5.09 μg/dL. **d**Compared with manganese quintiles 2–4. **e**Range, 28.14–73.95 μg/L.						

**Figure 1:**
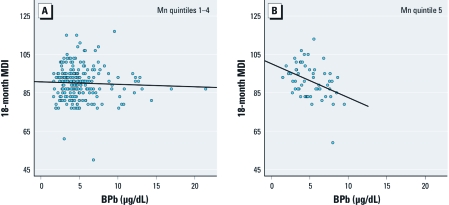
Scatterplots and regression lines of 12-month blood lead (BPb) and 18-month MDI among children with 12-month blood manganese (Mn) in quintiles 1–4 (*A*) and quintile 5 (*B*).

We observed no manganese–lead interaction among children in the lowest quintile of 12-month blood manganese [Table 2; adjusted β = –0.31 (95% CI: –1.25, 0.62)]. Therefore, we collapsed quintiles 1–4 and compared the association between lead and MDI in the highest manganese quintile to that in the lower four manganese quintiles. Figure 1 shows a steeper regression line for the association of lead with 18-month MDI among children in manganese quintile 5 versus quintiles 1–4, providing evidence for increased lead toxicity in the presence of high manganese levels [lead × quintile 5 manganese, adjusted β = –1.70 (95% CI: –2.95, –0.44)].

Twenty-four–month blood metals. We fit regression models for 24-month blood metals with 24-, 30-, and 36-month MDI separately, as well as in repeated measures models, but observed no significant manganese–lead interaction at either low or high manganese (Table 2).

*Associations of coexposure with psychomotor development.* Twelve-month blood metals. From repeated measures models, PDI scores are expected to decline by 0.27 points per 1-μg/dL increase in lead among children with midrange manganese levels, compared with a decline of 1.24 points (i.e., β_manganese quintile 5_ + β_lead_ + β_manganese quintile 5 × lead_ = –0.05 + –0.27 + –0.92) per 1-μg/dL increase in lead among children with high manganese levels (Table 2). This suggests more severe adverse effects of lead on psychomotor development among children with high manganese. Lower PDI scores were also observed in separate models at 18, 24, 30, and 36 months among children with a 1-μg/dL increase in 12-month lead and high manganese coexposure, compared with children with a 1-μg/dL increase in lead but only midrange manganese levels (Table 3). Effect estimates were consistent from 18 to 36 months and were statistically significant at 24, 30, and 36 months [24 months, adjusted β = –1.60 (95% CI: –2.93, –0.27); 30 months, adjusted β = –1.69 (95% CI: –3.21, –0.17); 36 months, adjusted β = –1.92 (95% CI: –3.56, –0.28)]. Although individual interaction effect estimates appeared to become stronger over time, the three-way interaction term lead × highest quintile of manganese × time) in the mixed-effects model did not reach statistical significance [β = –0.31 (95% CI: –0.64, 0.02)].

**Figure 2:**
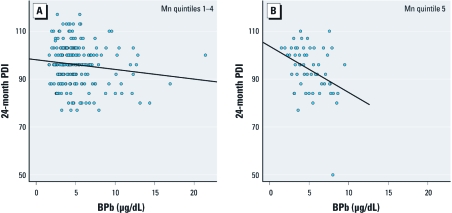
Scatterplots and regression lines of 12-month blood lead (BPb) on 24-month PDI among children with 12-month blood manganese (Mn) in quintiles 1–4 (*A*) and quintile 5 (*B*).

At low blood manganese levels, we did not observe a manganese–lead interaction. Figure 2 shows increased sensitivity to adverse lead effects on 24-month PDI among children with blood manganese in quintile 5 versus quintiles 1–4 [lead × highest manganese quintile, adjusted β = –1.57 (95% CI: –2.89, –0.25)].

Twenty-four–month blood metals. As with mental development, we observed no significant interactions between 24-month manganese and lead on psychomotor development (Table 2).

*Sensitivity analyses.* Crude estimates for 12-month manganese–lead interaction among all children, including those missing covariate data, were weaker than those for study participants with complete data but still suggest lower neurodevelopment scores with coexposure [e.g., crude β for interaction between manganese quintile 5 × continuous lead, 18-month MDI: all children, –0.96 (95% CI: –1.86, –0.06); children with complete data, –1.72 (95% CI: –2.95, –0.49); 30-month PDI; all children, –0.54 (95% CI: –1.64, 0.56); children with complete data, –1.74 (95% CI: –3.23, –0.25)]. In the subset of children who provided blood samples at both 12 and 24 months (*n* = 267 vs. total *n* = 275 for 12-month samples), results from 12-month metal analyses were similar [e.g., mixed-effects model adjusted β, manganese quintile 5 × continuous lead predicting MDI: subset of 267 children, –1.26 (95% CI: –2.17, –0.37); total sample, –1.27 (95% CI: –2.18, –0.37)]. For 24-month metal analyses, results for MDI among the subset of 267 children with both 12- and 24-month blood samples were generally consistent with results based on all 24-month samples [*n* = 437; mixed-effects model adjusted β, manganese quintile 5 × continuous lead: subset of 267 children, –0.53 (95% CI: –1.41, 0.35); total sample, –0.09 (95% CI: –0.63, 0.45)]. For PDI, however, we observed a negative interaction between 24-month lead and manganese only among the subset of children with both 12- and 24-month blood samples [mixed-effects model adjusted β, manganese quintile 5 × continuous lead: subset, –0.86 (95% CI: –1.66, –0.05); total sample, 0.18 (95% CI: –0.32, 0.68)]. When we excluded six outliers of 12-month blood levels (three highest manganese and lead levels) from analyses, effect estimates for the interaction between lead and high manganese on neurodevelopment were similar [e.g., mixed-effects model adjusted β, MDI: excluding outliers, –1.39 (95% CI: –2.36, –0.41); total sample, –1.27 (95% CI: –2.18, –0.37); PDI: excluding outliers, –1.00 (95% CI: –1.90, –0.10) ; total sample, –0.92 (95% CI: –1.76, –0.09)]. We also conducted analyses excluding one participant who had very low MDI and PDI scores at 24 months (scores of 50 for both), and results were similar, with the exception of a weaker effect estimate for 24-month PDI [adjusted β, manganese quintile 5 × continuous lead: excluding participant, –1.01 (95% CI: –2.32, 0.30); total sample, –1.60 (95% CI: –2.93, –0.27)].

## Discussion

This study suggests that joint exposure to manganese and lead is associated with greater deficits in both mental and psychomotor development than expected based on effects of exposure to either metal alone. Interestingly, this interaction was observed for 12-month but not for 24-month blood metal concentrations. A possible interpretation of the specificity of the interaction of 12-month blood manganese–lead levels is that this is a sensitive developmental window specific to this interaction. From repeated measures models, for a one-unit increase in lead at 12 months, MDI scores were 2.16 points lower among children with high manganese (–0.89 + –0.07 + –1.27 = –2.23), compared with children with midrange manganese [–0.07 (95% CI: –0.39, 0.25)]. PDI scores for a one-unit increase in lead at 12 months were 0.97 points lower among children with high manganese (–0.05 + –0.27 + –0.92 = –1.24), compared with children with midrange manganese [–0.27 (95% CI: –0.56, 0.02)]. Although these differences represent a small decrease in MDI and PDI scores for an individual, they may have large implications for the population level, because the entire distribution of neurodevelopment scores may shift, increasing the number of children with neurodevelopmental deficits that are of clinical concern.

The association between lead and Bayley scores was similar among children with low versus midrange manganese levels (i.e., no evidence of interaction). Although children in the lowest quintile of blood manganese had lower 12- and 18-month MDI scores than did children in quintiles 2–4, this association was observed only in the absence of a change in blood lead [manganese quintile 1, adjusted β = –2.86 (95% CI: –5.52, –0.21) and –3.36 (95% CI: –5.98, –0.74) at 12 and 18 months, respectively]. An independent effect of low manganese is consistent with its function as an essential nutrient, because manganese is a key component of various metalloenzymes needed for normal central nervous system function and protection against oxidative injury (Aschner and Aschner 1991). The lack of an interaction with lead at low manganese levels may be because the mechanisms by which manganese deficiency causes adverse effects may not overlap with those for lead, unlike manganese toxicity. Alternatively, the lowest levels observed in our population may not be low enough to produce an effect.

In another epidemiologic study that examined manganese–lead coexposure, Kim et al. (2009) reported significant inverse associations of lead with full-scale and verbal IQ among school-age children with manganese concentrations > 14 μg/L but not among children with levels < 14 μg/L. Although manganese and lead concentrations were lower than in our study [mean levels of 1.7 μg/dL lead and 14.3 μg/L manganese in Kim et al. (2009) vs. 5.0 μg/dL lead and 21.5 μg/L manganese for 24-month blood metals in the present study], the results are consistent across the two studies. An interaction between manganese and lead has also been seen in laboratory studies. In adult rats, exposure to manganese and lead resulted in synergistic effects on *in vivo* dopamine release (Rodriguez et al. 1998). In other laboratory studies, manganese exposure potentiated lead effects on learning and behavioral patterns and increased accumulation of metals in rat brains (Chandra et al. 1981, 1983; Shukla and Chandra 1987). Kalia et al. (1984) proposed that the presence of excess manganese in the brain may increase the binding affinity of brain proteins to lead or may induce expression of a lead-binding protein, resulting in more toxicity than expected based on effects of exposure to lead alone.

A direct comparison of manganese levels in our study with other studies is not possible because, to our knowledge, our study is the first to measure blood manganese in 12- and 24-month-olds. The average blood manganese levels among our participants are lower than average cord blood manganese reported previously by Takser et al. (2003) (38.5 μg/L) and by Zota et al. (2009) (40 μg/L) and higher than levels reported in 10-year-old children (12.8 μg/L) (Wasserman et al. 2006). This is consistent with evidence of declining manganese with increasing age (Chan et al. 1992; Collipp et al. 1983; Spencer 1999).

Our previous work, which reported lower MDI scores among children with both low and high manganese after adjusting for lead, found the strongest manganese associations at 12 months (Claus Henn et al. 2010). This association was not seen at later ages, suggesting a diminution over time. When lead and manganese exposures occur jointly at 12 months; however, the detrimental impact may be more enduring, because we see decrements in Bayley scores of similar magnitude at all ages studied. When results from both of our studies are taken together, it appears that, although blood manganese is associated with deficits in concurrent 12-month neurodevelopment test scores, a subset of children with lead coexposure have adverse manganese effects that persist. Because environmental manganese and lead frequently occur together in urban and/or contaminated areas (Aelion et al. 2009; Joselow et al. 1978), these results, if confirmed in other studies, may have implications for including the joint impact of chemicals in risk assessment and public health interventions rather than focusing on the independent effects of individual toxicants.

*Limitations.* We did not conduct a formal exposure assessment of these children and are thus unable to identify sources of metals exposure. However, blood lead levels among children residing in Mexico City during the time of this study (1997–2000) have been associated most strongly with the use of leaded gasoline and lead-glazed ceramics (Romieu et al. 1995). Elevated blood manganese levels likely arise from exposure to manganese in air, drinking water, or dietary sources, but differences in manganese levels among children with the same environmental exposures may also arise from variability in metabolic function that alters retention/excretion of internal manganese. Although it is unclear which biological matrix best represents manganese exposure, blood manganese is believed to be a reasonable indicator of environmental exposure and body burden (Agency for Toxic Substances and Disease Registry 2008).

Bayley scores may seem low relative to the expected mean score of 100 in a standard U.S. population. However, the BSID-IIS used in this study are not normalized to the Mexican population. Scores should therefore not be interpreted as low or be compared with other populations. Test results are internally valid and may be used to compare scores within this population but are not generalizable to other populations, which is a limitation of the study.

We lack data on socioeconomic status (SES) and quality of the home environment, two potential confounders of the relationship between manganese–lead and neurodevelopment. In the context of the present study, if social environment is associated with lead–manganese exposure and development, it may confound the “interaction” of these two metals. However, adjusting for maternal education, which is strongly correlated with SES among families in Mexico (Bronfman et al. 1988), likely addresses, at least in part, potential confounding by this variable. Furthermore, both SES and home environment are social influences that may also represent exposure opportunity. If these exposures and these variables are intertwined, adjusting for SES or home environment may mask the complete effects of exposure and may be inappropriate (Bellinger 2008b, 2009). Nonetheless, unmeasured and residual confounding remains possible. We also lack data on prenatal blood metals concentrations, which prevented us from assessing whether exposure before birth is more predictive of neurodevelopment in early childhood.

There may be concerns about selection bias due to the imbalance in the numbers of children for whom blood samples were available. Although some children provided only one sample whereas others provided two, we found no difference in major characteristics of these two groups (data not shown). However, we did observe a difference between 24-month metals interaction effect estimates for PDI based on all children with a 24-month sample and those based on children with both a 12- and 24-month sample. Although no differences in effect estimates were observed for MDI or for 12-month metals, we cannot exclude the possibility of a systematic difference between children who provided one blood sample versus two. This may also be a chance finding. Bias due to differential loss to follow-up is unlikely to alter conclusions because the attrition rate was low (9%) over the 2-year follow-up period. Effect estimates appear fairly robust, because interaction coefficients remained stable after excluding outliers, and crude estimates changed only minimally after controlling for sex, hemoglobin, gestational age, maternal IQ, and maternal education [for crude estimates, see Supplemental Material, Table 2 (http://dx.doi.org/10.1289/ehp.1003300)].

*Strengths.* This is the first prospective epidemiologic study to examine manganese–lead coexposure and neurodevelopment. With blood metal measurements at two time points in early childhood, a period of rapid brain development and potentially increased sensitivity, we were able to begin to assess effects of coexposures during critical windows of development. In addition, our repeated measurements of neurodevelopment between 12 and 36 months allowed us to evaluate persistence of associations and to fit repeated measures models with greater statistical power than is possible with cross-sectional analyses. Finally, our study population was stable, with little attrition and relative homogeneity in demographics that could act as confounders.

## Conclusion

We observed evidence of increased lead toxicity among young children with high manganese coexposure. Our study findings highlight the importance of understanding health effects of mixed exposures, particularly during potentially sensitive life stages of development such as early childhood. Future research should emphasize coexposures to these neurotoxicants in other populations examined prospectively.

## Supplemental Material

(135 KB) PDF
