# Supplementary material for: Associations of Early Childhood Manganese and Lead Coexposure with Neurodevelopment
Source: Environ Health Perspect. 2011 Sep 1;120(1):126–31. doi: 10.1289/ehp.1003300 (PMC3261931; doi:10.1289/ehp.1003300)
Supplement: (135 KB) PDF [file ehp.1003300.s001.pdf]

Supplemental Material

Title: Associations of Early Childhood Manganese and Lead Co-exposure with Neurodevelopment

Author names: Birgit Claus Henn[1], Lourdes Schnaas[2], Adrienne S. Ettinger[1,3], Joel Schwartz[1,4], Héctor Lamadrid-Figueroa[5], Mauricio Hernández-Avila[6], Chitra Amarasiriwardena[4], Howard Hu[7], David C. Bellinger[1,8], Robert O. Wright[1,4,7,9]*, Martha María Téllez-Rojo[5]*

Supplemental Material, Table 1. Sociodemographic Characteristics of Study Participants Included in Analyses of 12- and 24-Month Blood Metals

	Children Included in Analyses of:	
	12 Month Metals[a]	24 Month Metals[b]
**Characteristics**	**Mean (SD) or %**	**Mean (SD) or %**
Sex (male, %)	51.3%	51.0%
Birth weight (kg)	3.1 (0.4)	3.1 (0.4)
Estimated gestational age (weeks)	38.9 (1.3)	38.9 (1.3)
Birth length (cm)	49.9 (2.2)	49.8 (2.3)
Head circumference (cm; delivery)	34.1 (1.5)	34.1 (1.6)
Blood manganese (μg/l)		
12-month	24.7 (5.9)	24.8 (6.0)[e]
24-month	19.2 (6.8)[c]	21.5 (7.4)
Blood lead (μg/dl)		
Umbilical cord	4.2 (2.7)[d]	4.7 (3.2)[f]
12-month	5.1 (2.6)	5.1 (2.6)[e]
24-month	4.8 (2.5)[c]	5.0 (2.9)
Hemoglobin (g/dl)		
12-month	11.9 (1.3)	11.8 (1.3)
24-month	12.6 (1.2)	12.6 (1.1)
Bayley MDI Score (36-month)[g]	92.3 (9.1)	93.3 (9.1)
Bayley PDI Score (36-month)[g]	94.9 (10.8)	95.9 (10.9)
Maternal marital status (% married)	70.9%	72.4%
Maternal IQ	87.8 (12.6)	88.1 (13.1)
Maternal education (years)	10.7 (2.7)	10.7 (2.8)
Maternal age at delivery (years)	25.6 (5.3)	25.9 (5.3)
Maternal whole blood lead (μg/dl)[h]	6.9 (4.2)	7.5 (4.6)

a – Sample size is 275, except where noted b – Sample size is 437, except where noted
c – n=266 d – n=183 e – n=267 f – n=278
g – n=250. Only 36-month Bayley scores provided for brevity; scores at other time points are similar h – Collected at 1-month postpartum

Supplemental Material, Table 2. Crude Results from Mixed Models of 12- and 24-month Metals with Repeated Measures of Bayley Scores[a]

MDI:

Exposure	Effect Estimates for 12-month Metals[b] Beta	(95% CI)	Effect Estimates for 24-month Metals[c] Beta	(95% CI)
Manganese (quintile 1[d] vs. quintiles 2-4)	-2.15	(-4.14 to -0.16)	-0.08	(-2.19 to 2.03)
Manganese (quintile 5[e] vs. quintiles 2-4)	-0.86	(-2.82 to 1.11)	0.43	(-1.65 to 2.51)
Lead (μg/dl)[f]	-0.17	(-0.50 to 0.16)	-0.37	(-0.76 to 0.03)
Manganese (quintile 1) * lead	-0.47	(-1.45 to 0.51)	0.21	(-0.80 to 1.22)
Manganese (quintile 5) * lead	-1.38	(-2.32 to -0.45)	0.15	(-0.41 to 0.71)

PDI:

Exposure	Beta	(95% CI)	Beta	(95% CI)
Manganese (quintile 1 vs. quintiles 2-4)	0.53	(-1.23 to 2.30)	-0.21	(-2.07 to 1.65)
Manganese (quintile 5 vs. quintiles 2-4)	-0.31	(-2.05 to 1.42)	0.19	(-1.64 to 2.01)
Lead (μg/dl)	-0.32	(-0.61 to -0.02)	-0.29	(-0.64 to 0.05)
Manganese (quintile 1) * lead	0.004	(-0.87 to 0.88)	0.32	(-0.57 to 1.21)
Manganese (quintile 5) * lead	-0.96	(-1.79 to -0.13)	0.27	(-0.22 to 0.77)

a – Effect estimates for 12-month metals are from repeated measures models that include Bayley scores at 12, 18, 24, 30, and 36 months. Effect estimates for 24-month metals are from repeated measures models that include Bayley scores at 24, 30, and 36 months.

b – Number of subjects = 275. For MDI, number of observations = 1330; for PDI, number of observations = 1329

c – Number of subjects = 437. For MDI and PDI, number of observations = 1240

d – Blood manganese levels in quintile 1 range from 15.30 to 20.16 µg/l at 12-months; 5.06 to 15.57 µg/l at 24-months

e – Blood manganese levels in quintile 5 range from 28.14 to 73.95 µg/l at 12-months; 25.78 to 93.53 µg/l at 24-months

f – Blood lead centered at mean (12-months: 5.09 µg/dl; 24-months: 4.98 µg/dl)
